# Clinical significance and immune landscape of a novel ferroptosis-related prognosis signature in osteosarcoma

**DOI:** 10.1186/s12885-023-10688-7

**Published:** 2023-03-10

**Authors:** Liyu Yang, Jiamei Liu, Shengye Liu

**Affiliations:** 1 Department of Orthopedics, The Shengjing Hospital of China Medical University, Shenyang, Liaoning 110004 People’s Republic of China; 2 Department of Pathology, The Shengjing Hospital of China Medical University, Shenyang, Liaoning 110004 People’s Republic of China

**Keywords:** Osteosarcoma, Ferroptosis, Signature, Immune, Prognosis

## Abstract

**Background:**

Osteosarcoma is a malignant tumor that usually occurs in adolescents aged 10–20 years and is associated with poor prognosis. Ferroptosis is an iron-dependent cell death mechanism that plays a vital role in cancer.

**Methods:**

Osteosarcoma transcriptome data were downloaded from the public database TARGET and from previous studies. A prognostic risk score signature was constructed using bioinformatics analysis, and its efficacy was determined by analyzing typical clinical features. The prognostic signature was then validated with external data. Differences in immune cell infiltration between high- and low-risk groups were analyzed. The potential of the prognostic risk signature as a predictor of immunotherapy response was evaluated using the GSE35640 (melanoma) dataset. Five key genes expression were measured by real-time PCR and western blot in human normal osteoblasts and osteosarcoma cells. Moreover, malignant biological behaviors of osteosarcoma cells were tested by modulating gene expression level.

**Results:**

We obtained 268 ferroptosis-related genes from the online database FerrDb and published articles. Transcriptome data and clinical information of 88 samples in the TARGET database were used to classify genes into two categories using clustering analysis, and significant differences in survival status were identified. Differential ferroptosis-related genes were screened, and functional enrichment showed that they were associated with HIF-1, T cells, IL17, and other inflammatory signaling pathways. Prognostic factors were identified by univariate Cox regression and LASSO analysis, and a 5-factor prognostic risk score signature was constructed, which was also applicable for external data validation. Experimental validation indicated that the mRNA and protein expression level of MAP3K5, LURAP1L, HMOX1 and BNIP3 decreased significantly, though meanwhile MUC1 increased in MG-63 and SAOS-2 cells compared with hFOB1.19 cells. Cell proliferation and migration ability of SAOS-2 were affected based on alterations of signature genes.

**Conclusions:**

Significant differences in immune cell infiltration between high- and low-risk groups indicated that the five ferroptosis-related prognostic signature was constructed and could be used to predict the response to immunotherapy in osteosarcoma.

**Supplementary Information:**

The online version contains supplementary material available at 10.1186/s12885-023-10688-7.

## Background

Osteosarcoma is a malignant tumor originating from mesenchymal tissue that accounts for 20% of primary malignant bone tumors. It occurs in the epiphysis of blood-rich bone tissue in children and adolescents and has a very poor prognosis, causing significant physical and psychological trauma in patients [1]. The combination of neoadjuvant chemotherapy (NACT), surgical resection, and adjuvant chemotherapy is currently the accepted treatment in China and abroad [2]. However, the 5-year survival rate for patients with osteosarcoma remains at 60–70%. Many patients exhibit chemoresistance, and the high metastatic and high recurrence rates of osteosarcoma have not been addressed by increasing doses or optimizing treatment regimens [3], resulting in nearly half of the patients ultimately declaring treatment failure [4].

The identification of tumor prognostic signatures and the characterization of relevant molecules have led to considerable progress in our understanding of the responses to treatment in malignant tumors [5, 6]. Therefore, comprehensively investigating the pathogenesis of osteosarcoma is essential to construct effective prognostic signatures and find potential therapeutic targets for guiding clinical treatment decisions in osteosarcoma.

Ferroptosis is a form of cell death caused by the loss of intracellular iron-dependent glutathione peroxidase activity and lipid peroxide accumulation, and mitochondria play a key role in the process of cellular ferroptosis [7, 8]. Ferroptosis is a novel form of programmed cell death that differs from cell necrosis, autophagy, and apoptosis, and plays a role in diseases such as ischemic organ injury and cancer. Ferroptosis inducers such as sorafenib are currently in clinical use, and several studies have shown that ferroptosis-related mechanisms may be useful for the design of cancer treatments [9, 10]. It has become a breakthrough point in tumor treatment in recent years. Ferroptosis is closely related to the occurrence, progression, and prognosis of osteosarcoma, as well as its sensitivity to chemotherapy [11–14]. Here, we constructed a risk score signature based on five ferroptosis-related genes for the prognostic risk classification of osteosarcoma.

Previous studies have revealed that ferroptosis and immune cell infiltration are closely correlated with the development and progression of cancer [15–17]. However, no systematic investigation or research has been conducted to elucidate the communicative functions of ferroptosis and immune infiltration in osteosarcoma mechanisms, we comprehensively analyzed the potential mechanisms of ferroptosis-related hub genes and immune infiltration cells in osteosarcoma, which has rarely been the foci of prior studies. Significant differences in immune infiltration were found between the different risk groups based on ferroptosis-related risk score signature. The efficacy of immunotherapy for the treatment of cancer has been demonstrated, and its indications are continually increasing, including osteosarcoma [18, 19]. Based on the theory above, we used the ferroptosis-related signature to identify targets of immunotherapy for osteosarcoma.

## Materials and methods

### Data download and processing

Transcriptome data were downloaded from the public database TARGET (https://xenabrowser.net/datapages/), which contains 88 samples of osteosarcoma, including clinical information such as age, gender, stage, survival time, and presence or absence of metastasis. Gene expression profile data were obtained by gene annotation. Ferroptosis-related genes were identified using the online database FerrDb (http://www.zhounan.org/ferrdb/index.html) and published articles [9, 10].

### Clustering identification and differential expression analysis

Based on the ferroptosis-related gene set, the non-negative matrix factorization (NMF) method was used to cluster the samples into two categories. R package Survminer was used to evaluate prognosis. T-test differential expression analysis was used to filter differentially expressed genes between clusters.

### Construction of a prognostic risk score signature

Univariate Cox regression analysis was used to identify prognostic factors based on ferroptosis-related genes with inter-cluster differences, followed by least absolute shrinkage and selection operator (LASSO) analysis to remove redundant factors. The prognostic risk score signature was generated, and typical clinical features were included to validate the prognostic risk score signature. The risk signature showed better predictive efficacy than clinical features such as age and gender.

### Efficacy of the prognostic risk score signature

The GEO dataset GSE21257 was used for external data validation of the prognostic risk score signature. Classification efficacy was assessed by prognostic survival analysis, ROC, AUC, and univariate and multivariate Cox regression analyses. Collograms were constructed with clinical features to demonstrate that it can be used as a clinical feature to classify samples.

### Evaluation of immunotherapy predictors in the prognostic risk score signature

Considering the immunological differences between the high- and low-risk ferroptosis factor-related clusters, differences in immune cell infiltration between the high- and low-risk groups were analyzed by CIBERSORT. The GSE35640 dataset (melanoma dataset) was used to evaluate whether the prognostic risk signature could be used as a predictor of immunotherapy, and efficiency was evaluated.

### Analysis of the prognostic risk signature combined with clinical characteristics

The Sankey diagram can intuitively show the proportion of patients with metastasis and those without metastasis in the high and low-risk groups. We constructed a Sankey chart by combining the high- and low-risk prognostic groups, clustering classification, and clinical characteristics.

### Biological experimental validation on quantification of gene expression by real-time PCR

Relative mRNA expression level of five genes were measured among hFOB1.19, MG-63 and SAOS-2 cell lines. Human osteoblast cell line hFOB1.19 was maintained in DMEM/F12 medium at 34^◦^C with 5% CO_2_ in a humidified atmosphere. Human osteosarcoma cell line MG-63 and SAOS-2 cells were cultured in MEM and Macoy’5A medium, respectively. Medium were all supplemented with a 10% FBS, 100 µg/ml streptomycin and 100 U/ml penicillin at 37^◦^C with 5% CO_2_ in a humidified atmosphere.

Total RNA was extracted with TRIzol reagent then used to synthesize cDNA using SuperScript II reverse transcriptase (Invitrogen; Thermo Fisher Scientific, Inc.) with 5 μg oligo (dT) primers per sample. By using SYBR Green PCR master mix (Applied Biosystems; Thermo Fisher Scientific, Inc.), qPCR was performed in a total volume of 20 μL in a 7500 Real-Time PCR System (Applied Biosystems; Thermo Fisher Scientific, Inc.) as follows: 95˚C for 5 min, and 40 cycles of 95˚C for 30 s and 60˚C for 45 s. Melt-curve analysis was used to confirm the specificity of the amplification and GAPDH served as the endogenous control for normalization of amount of total RNA in each group. The relative levels of gene expression were performed as ΔCq = Cq_gene_ – Cq_reference_, and the gene expression was calculated in fold change according to the 2^−ΔΔCq^ method while repeated independently in triplicate.

The primer sequences were designed as follows: forward, 5'-AGGGCTCCTGGGTAGAACT-3' and reverse, 5'-CTCCATTATAAATAGAAACCGAGGC-3' for BNIP3; forward, 5'-CACAGTGCTTACAGTTGTTACG-3' and reverse, 5'-TGGTCATACTCACAGCATTCTT-3' for MUC1; forward, 5'-GGAGAAAGAGATGTCAAGGGAA-3' and reverse, 5'-CAATTTTGTCTTGGTCTTCCGT-3' for MAP3K5; forward, 5'-CTGGACACGTTGGCGGATGATG-3' and reverse, 5'-CGCTTGTGTAGTGCCTGTGAGTC-3' for LURAP1L; forward, 5'-CCTCCCTGTACCACATCTATGT-3' and reverse, 5'-GCTCTTCTGGGAAGTAGACAG-3' for HMOX1; forward, 5'-TCAAGGCTGAGAACGGGAAG-3' and reverse, 5'-TGGACTCCACGACGTACTCA-3' for GAPDH.

### Biological experimental validation on quantification of gene expression by Western blot analysis

Total proteins from each well were harvested in ice-cold radioimmunoprecipitation (RIPA) lysis buffer (Thermo Fisher Scientific, Inc.) supplemented with phenylmethanesulfonyl fluoride for 1 h. The protein concentration was quantified by bicinchoninic acid protein assay kit (Sigma-Aldrich) according to the manufacturer’s instructions. Equal proteins of each treatment were separated on 12% sodium dodecyl sulfate polyacrylamide (SDS-PAGE) gels (Beyotime Institute of Biotechnology, Haimen, China) and electrophoretically transferred onto polyvinylidene difluoride (PVDF) membranes (Millipore, Bedford, MA, USA). The membranes were soaked in 5% skimmed milk as blocking buffer for 1 h, then washed in Tris-buffered saline Tween-20 [TBST;150 mmol/l NaCl (PH 7.5), 20 mmol/l Tris–HCl and 0.1% Tween 20] at room temperature for 3 times. The membranes were incubated with primary monoclonal antibodies against MUC1 (ab182560, abcam), MAP3K5 (ab131506, abcam), LURAP1L (LURAP1L antiody, Santa Cruz Biotechnology), HMOX1 (ab68477, abcam), and BNIP3 (ab109362, abcam) at 1:1000 dilution overnight at 4 °C followed by hybridized with horseradish perosidase (HRP)-conjugated secondary antibody (Santa Cruz Biotechnology) and visualized by using enhanced chemiluminescence as the HRP substrate. The relative protein levels were calculated based on β-actin (MD6553, MDL) as the loading control.

### Biological experimental validation on influence of five key gene expression on malignant biological behavior of osteosarcoma cells.

Small interfering RNA (siRNA) transfection methods were applied to knock down key gene expression. Key genes siRNA and a negative control (siRNA-NC) were purchased from GenePharma (Shanghai, China). Si-NC and siRNA were transfected into SAOS-2 cells cultured in six-well plates by using Lipofectamine 3000 (Invitrogen), following the manufacturer’s instructions. The knockdown effect of siRNA was measured by westernblot after transfected for 48 h as the manufacturer’s instructions. Cell viability was carried out using CCK-8 toolkit. The specific experimental procedure is described as previously. Relative cellular viability was recorded. Furthermore, wound healing assay was used to assess migration ability of osteosarcoma cells. SAOS-2 cells were transfected with siRNA in six-well plates and cultured until reaching 90% confluence. A wound was created with a pipette tip, the cells were washed twice, and cultured in medium without FBS. The wound was observed and photographed at 0 h and 24 h using an inverted microscope (Nikon, Japan). Cell migration ability was described as the number of cells that migrated into the wound. All assays were performed in triplicate. All the presented data and results were processed using GraphPad Prism 9 software and expressed as mean ± standard deviation of at least three independent experiments. T-test was used to determine statistical significance. *P < 0.05, **P < 0.01, ***P < 0.001, ****P < 0.0001 were considered to indicate statistically significant differences.

## Results

### Osteosarcoma transcriptome and ferroptosis-related gene processing

**Fig. 1:**
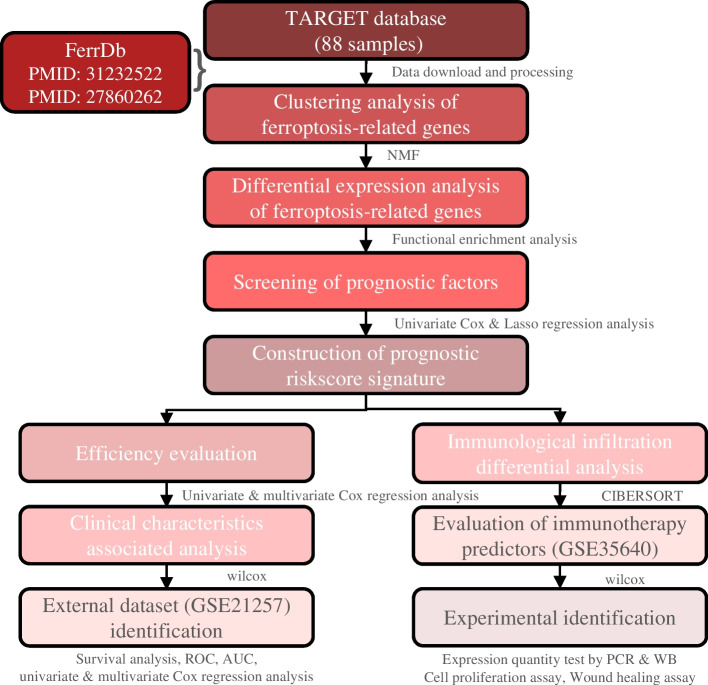
The workflow of this study

Osteosarcoma transcriptome data were retrospectively downloaded from the public database TARGET. A total of 88 samples were analyzed including clinical information such as age, gender, stage, survival time, and the presence or absence of metastasis. Gene expression profile data were obtained by gene annotation (Supplementary table 1). We identified 268 ferroptosis-related genes from the online database FerrDb and previous research (Supplementary table 2). The workflow of this study is displayed in Fig. 1.

### Clustering survival difference analysis based on the ferroptosis dataset

**Fig. 2:**
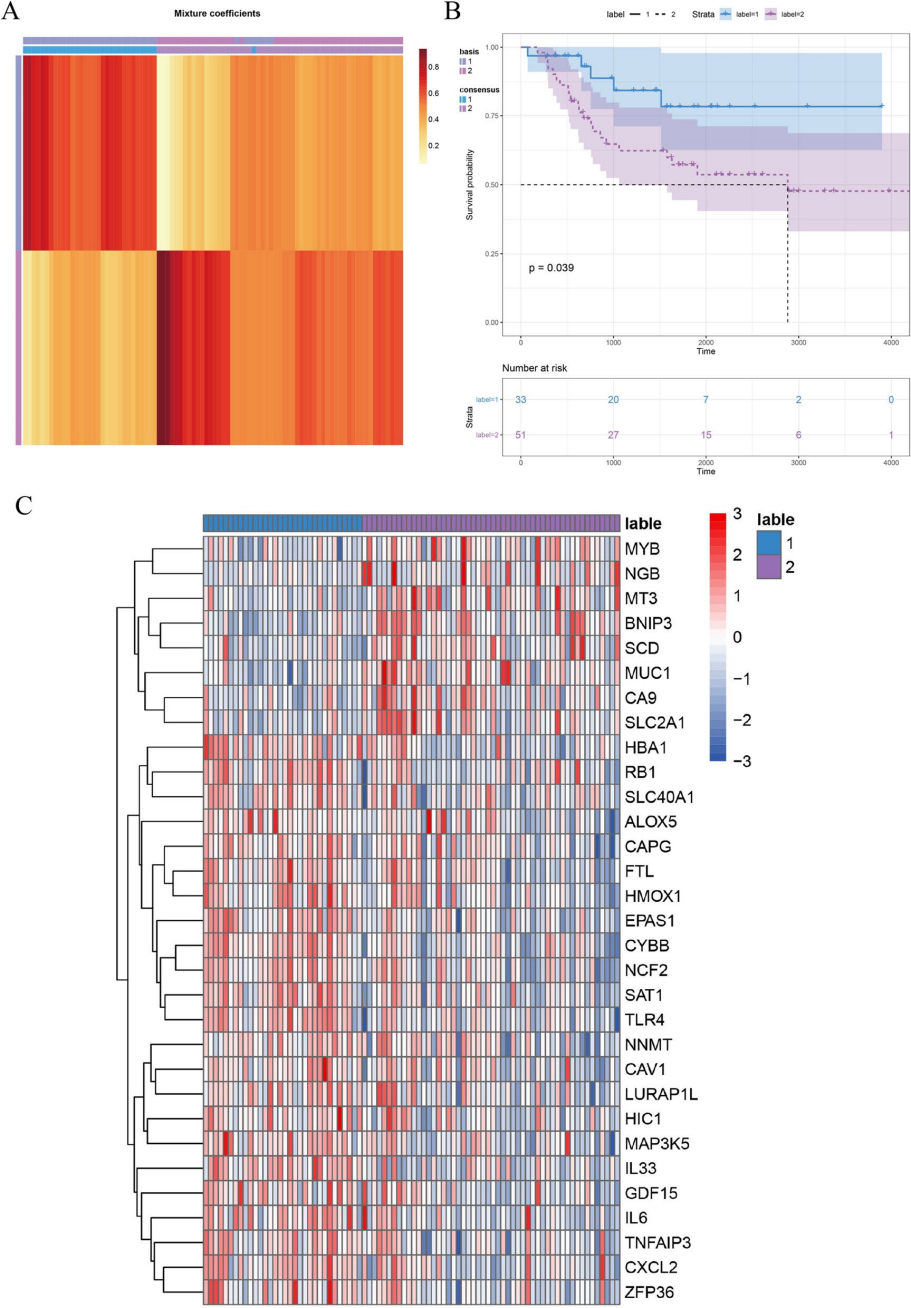
**A** NMF cluster analysis results. The horizontal axis represents the classification category. **B** Kaplan–Meier survival curve of two clusters. The horizontal axis represents survival time, and the vertical axis represents survival rate. **C** Heatmap of differentially expressed ferroptosis-related genes in the two lables. The horizontal and vertical axes represent the classification category, the vertical axis represents the gene, and the depth of color represents gene expression

The NMF method was used to cluster the samples into two categories based on the TARGET ferroptosis-related gene data (Fig. 2). The R package Survminer was used for survival prognosis, which showed a significant prognostic value for each cluster (*P* = 0.039). The R package Pheatmap was used to generate the heatmap.

### Ferr-DEG recognition and enrichment analysis

**Fig. 3:**
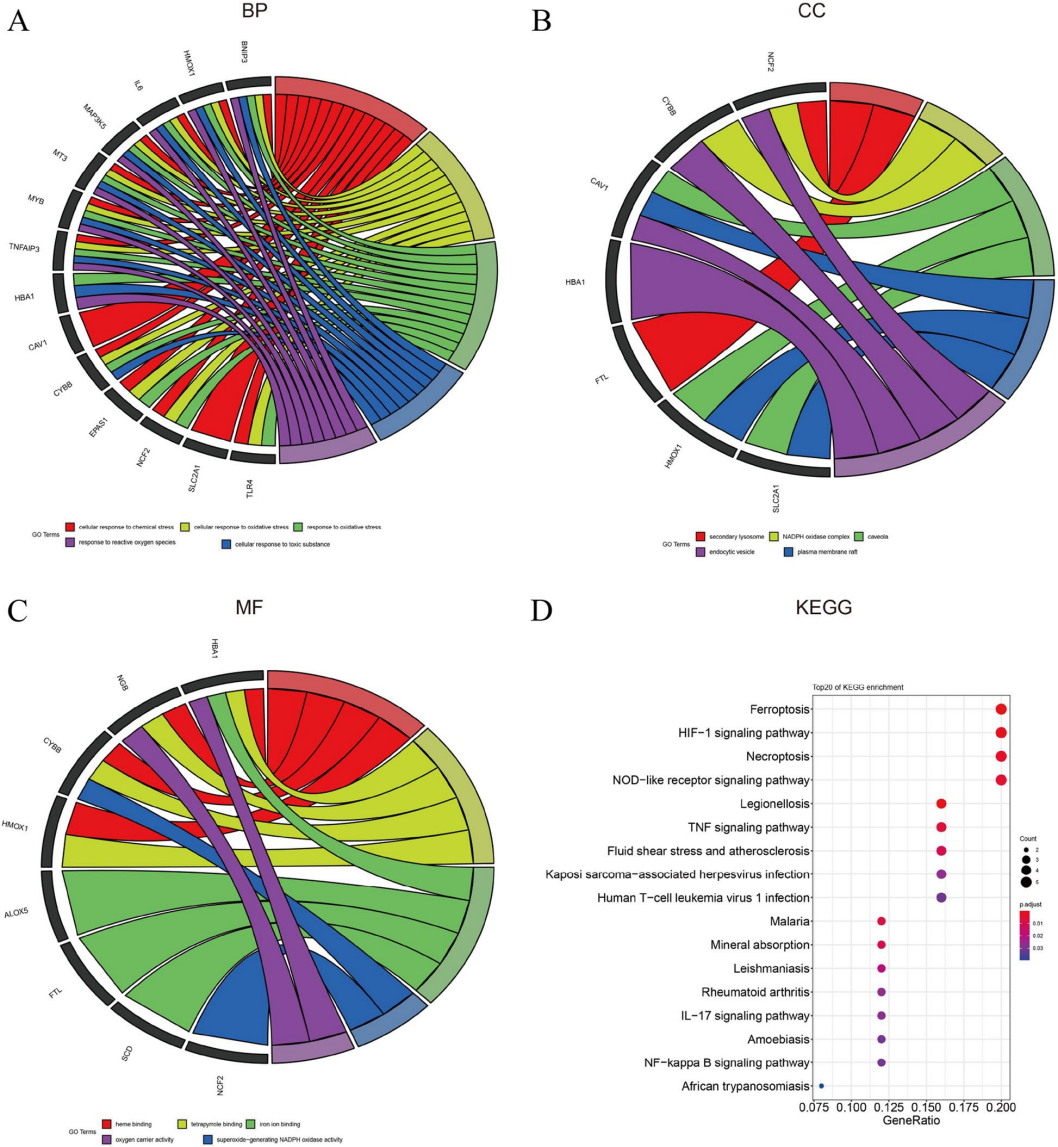
Enrichment analysis of differentially expressed ferroptosis-related genes

According to T-test differential expression analysis, 31 differentially expressed ferroptosis-related genes (**Ferr-DEGs**) between different clusters were screened (*P* < 0.05). Functional enrichment analysis indicated that these genes were associated with HIF-1, T cells, and IL17 inflammatory signaling pathways (Fig. 3).

### Identification of prognostic factors

**Fig. 4:**
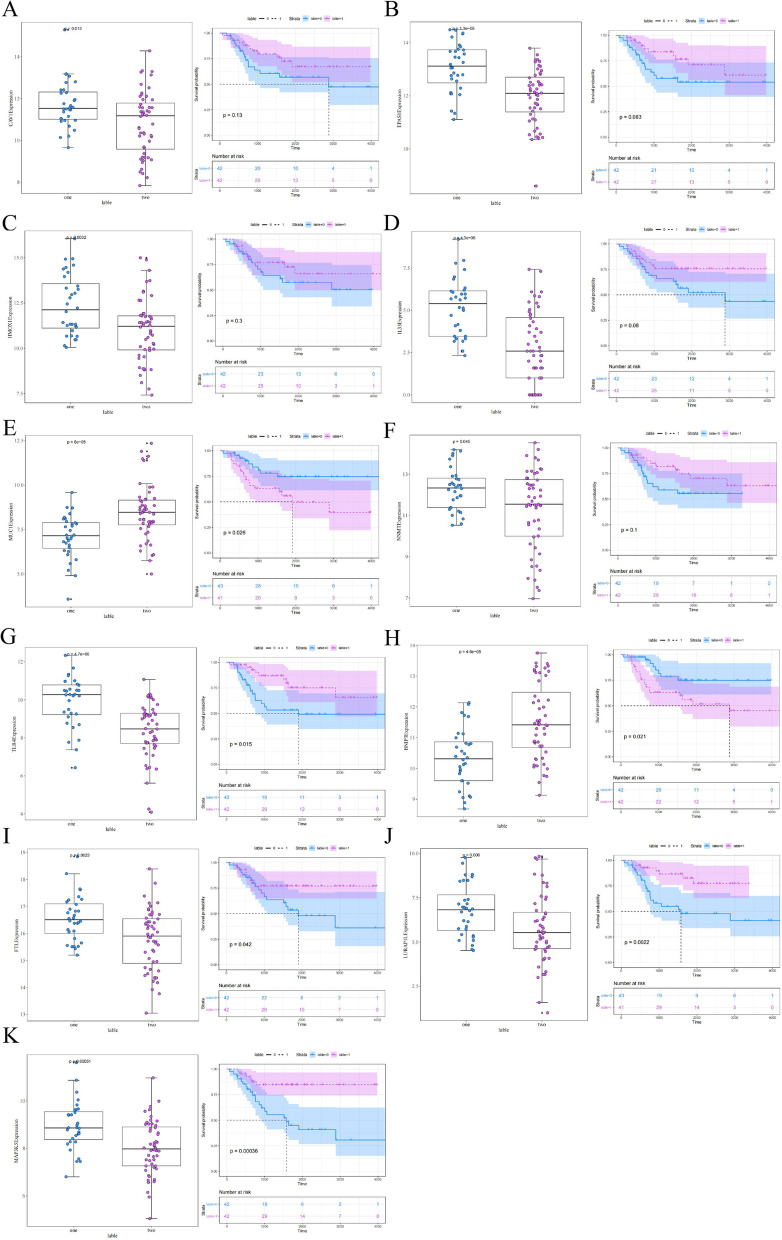
Differential expression and prognostic analysis of 11 ferroptosis-related genes

Univariate Cox regression analysis was performed to identify prognostic factors based on ferroptosis-related genes associated with inter-cluster differences. Eleven genes were screened out according to COX P-values < 0.05; the results of the prognostic analysis are shown in Fig. 4.

### Prognostic risk score signature construction 

**Fig. 5:**
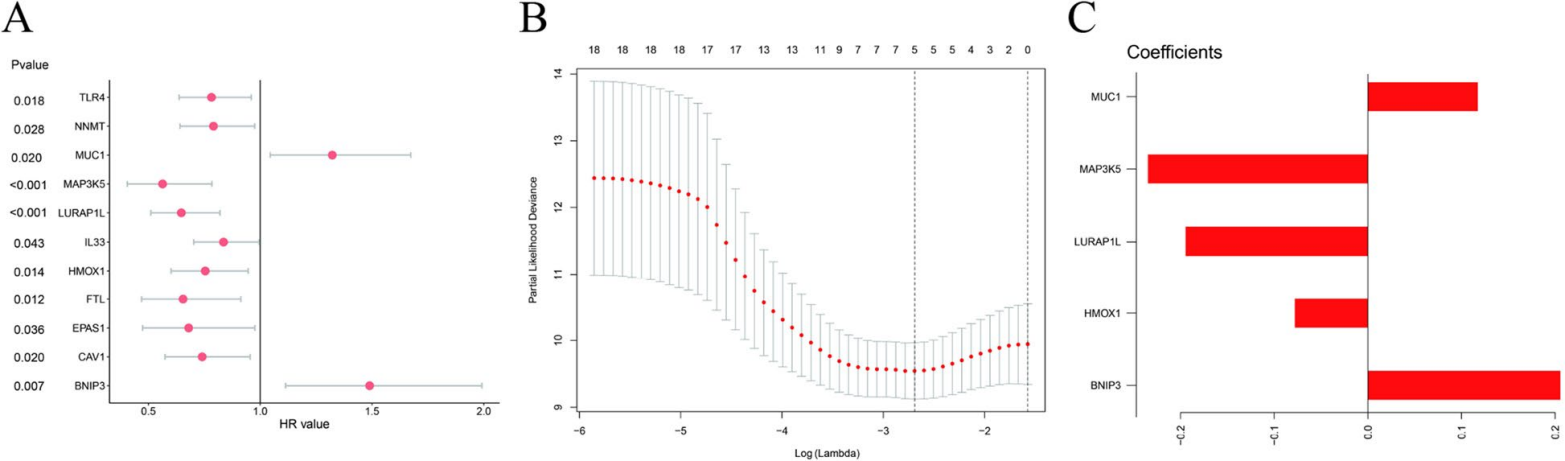
Prognostic risk score signature construction. **A** Among the 11 genes screened by Cox regression, the left side represents the *P* value of each gene obtained by Cox regression, the middle is the gene, and the right side represents the HR value obtained by Cox regression (> 1 indicates a risk factor, and < 1 indicates a protective factor). **B** LASSO regression results show the dotted line position as 5. Five genes were screened to build the signature. **C** Results obtained by LASSO regression show that the dotted line position points to 5. Five genes were finally screened to build the signature. The horizontal axis of the five genes screened by LASSO regression represents the LASSO regression coefficient of each gene, which is the coefficient of each gene in the risk score

LASSO regression analysis was used for dimension reduction to remove redundant factors, and five related factors were selected to build a prognostic risk score signature (Fig. 5). Risk score = 0.11735 × MUC1—0.23479 × MAP3K5—0.19464 × LURAP1L—0.07795 × HMOX1 + 0.20553 × BNIP3.

### Efficiency evaluation of the prognostic risk score signature and clinical characteristics 

**Fig. 6:**
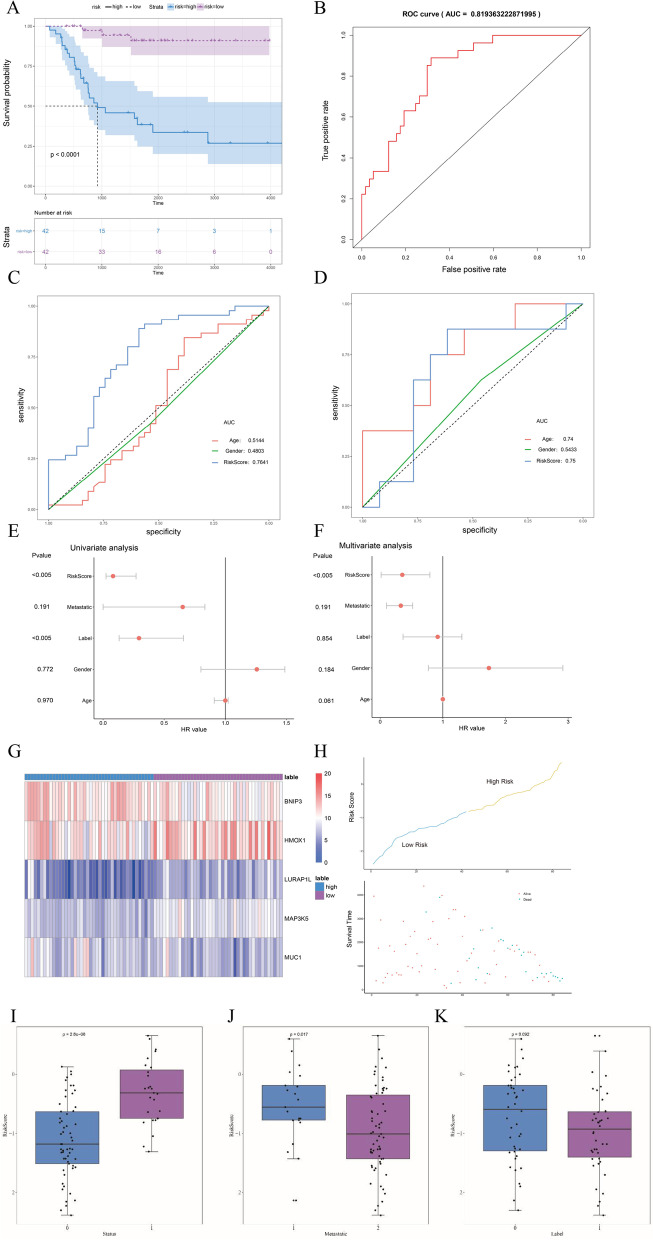
Effectiveness evaluation of the prognostic risk score signature (**A**-**D**). Survival curves for the high- and low-risk groups. The horizontal axis shows time and the longitudinal axis is survival probability, **B** ROC curve. The horizontal axis shows the false positive fraction and the longitudinal axis shows the true positive fraction. **C** AUC curve of clustering groups; the horizontal axis shows specificity and the longitudinal axis shows sensitivity. **D** AUC curve of metastatic grouping; the horizontal axis shows specificity and the longitudinal axis shows sensitivity. **E** Univariate Cox regression for all clinical indicators. The horizontal axis represents the HR value (greater than 1 is a risk factor, less than 1 is a protective factor), and the vertical axis represents the P value of single genes and univariate Cox regression. **F** Multivariate Cox regression results of clinical indicators. The horizontal axis represents the HR value (greater than 1 is a risk factor, less than 1 is a protective factor), and the vertical axis represents the P value of single genes and multivariate Cox regression. **G** Heatmap of differential expression of genes. The horizontal axis represents classification, the vertical axis represents genes, and the color represents expression. **H** Corresponding states of the sample survival time and the high- and low-risk groups. Differential display of risk score in clinical characteristics grouping (**I**-**K**). I. 0 refers to the state of alive or existence. 1 refers to the state of death. **J** 1 refers to the occurrence of metastasis and 2 refers to the absence of metastasis. **K** 0 refers to the first category of the consistent clustering, and 1 refers to the second category of the consistent clustering

The samples were classified into high- and low-risk groups according to the prognostic risk score signature, and prognostic survival analysis showed significant prognostic efficacy (****P < 0.0001). ROC curves and AUC were used to evaluate the efficacy. The results showed that the classification efficacy of the prognostic risk score signature was better than that of clinical characteristics such as age and gender, as shown in Fig. 6 (A-D).

Univariate and multivariate Cox regression analyses confirmed that the prognostic risk score signature had better classification efficacy than clinical characteristics such as age and gender (Fig. 6E, F).

The prognostic risk score signature was evaluated by applying typical clinical characteristics, which showed that it had better predictive efficacy than clinical characteristics such as age and gender (Fig. 6G, H). The Wilcoxon test was used to calculate the *P*-value with better predictive efficacy in clinical characteristics such as survival status, occurrence of metastasis, and category labeling (Fig. 6I-K).

### Validation of the prognostic signature in the external dataset 

**Fig. 7:**
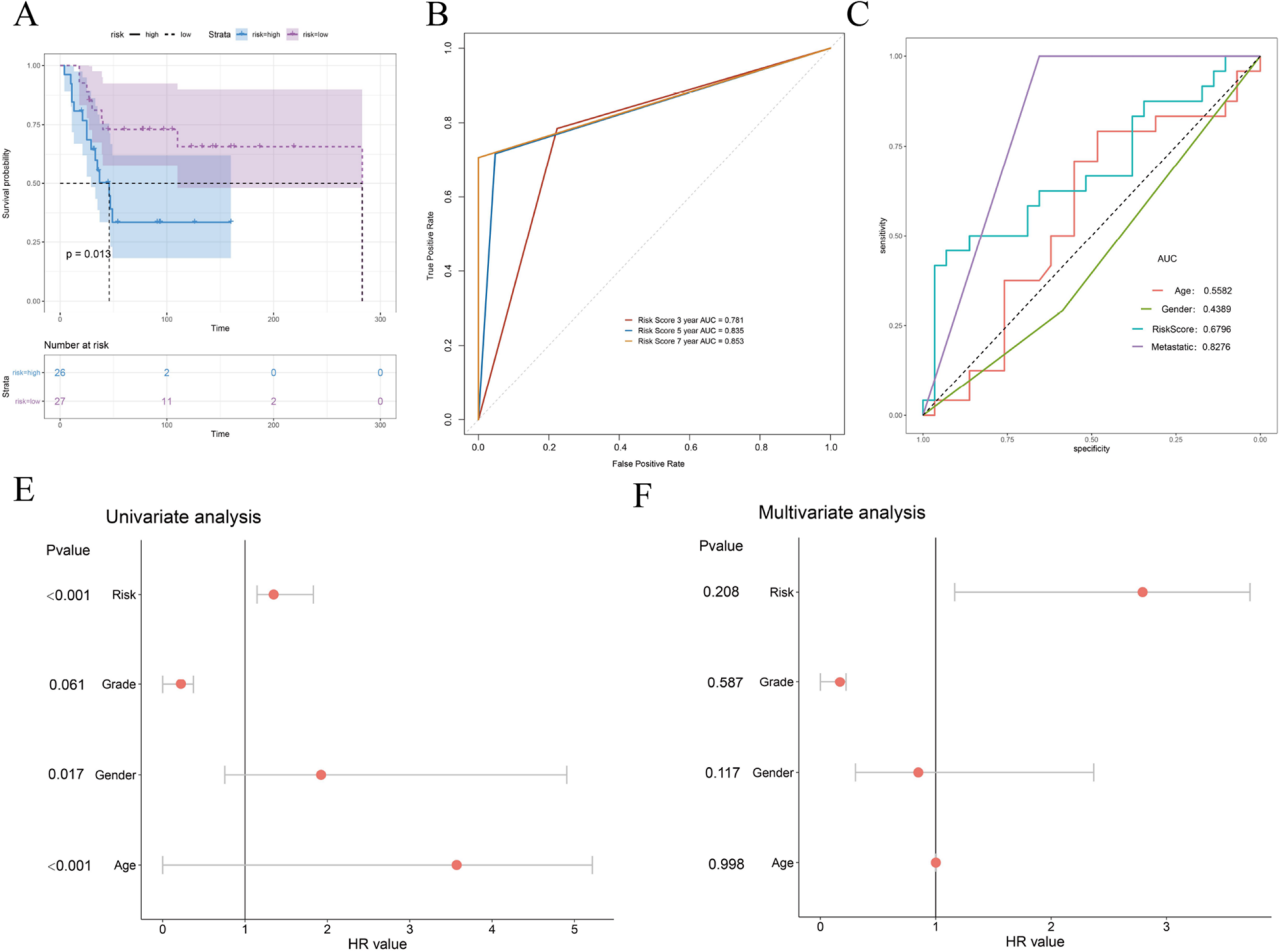
External dataset validation. **A** Survival analysis curve. **B** AUC curves for different survival times. **C** AUC curves for different clinical characteristics. **D** Univariate Cox regression analysis results. **E** Multivariate Cox regression results of multiple factors

The prognostic risk score signature was applied to the GEO dataset GSE21257 for external data validation (Fig. 7), including 53 samples (Supplementary table 4). Prognostic survival analysis, ROC, AUC, and univariate as well as multivariate Cox regression analyses showed that it had a good classification efficiency.

### Establishment of a columnar table of clinical characteristics 

**Fig. 8:**
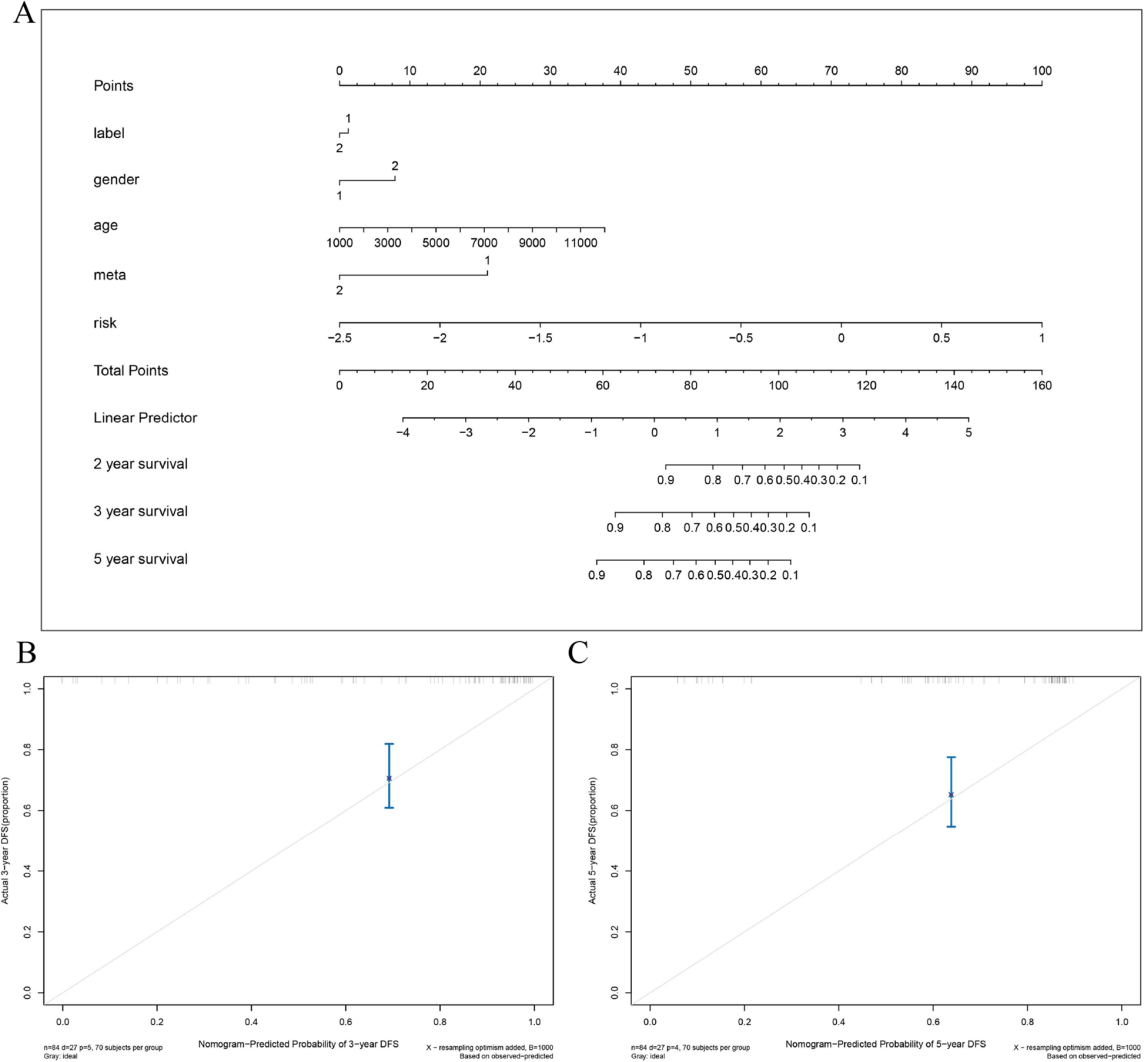
Nomograms for clinical features. **A** represents different clinical features in the classification effect. **B** and **C** show the calibration curves for the nomogram predicting 3-year and 5-year disease-free survival (DFS) rates

The columnar table of clinical characteristics shown in Fig. 8 was constructed by combining age, gender, clustering type, metastasis occurrence, and risk score.

### Immunological infiltration differential analysis

**Fig. 9:**
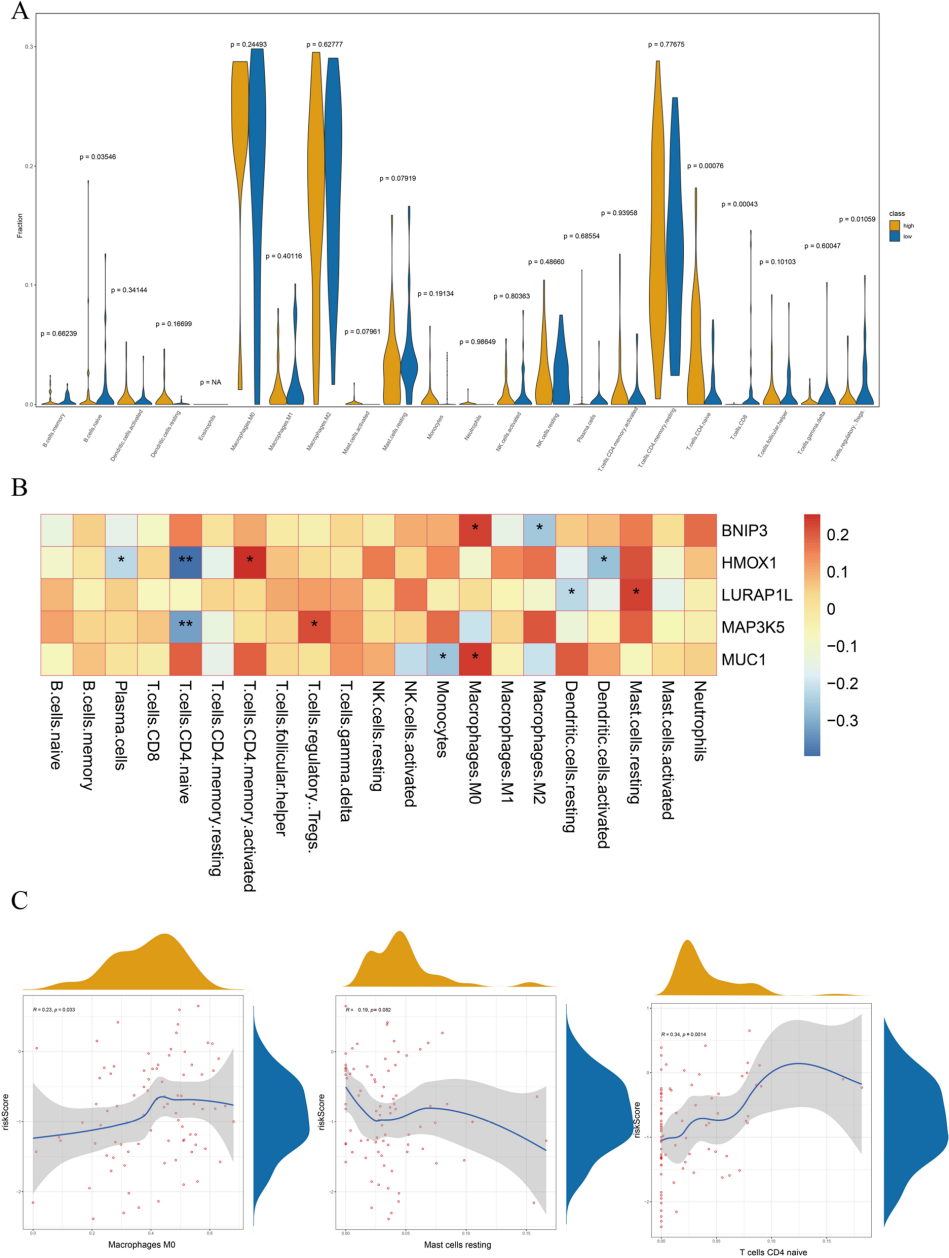
Differential analysis of immune infiltrating cells between high- and low-risk groups. **A** Differential state of immune infiltrating cells in different types. **B** Correlation between expression of five genes and immune cells infiltration state. **C** The diagrams for distribution of the risk score in three types of immune cells

Considering the immune differences between the high- and low-risk groups of ferroptosis factors, we performed differential analysis of immune cell infiltration based on high and low prognostic risk groups, and significant differences in infiltration were found in subtypes of B cells and some T cells by the Wilcox test (Fig. 9).

### Evaluation of immunotherapy predictors using the prognostic risk score signature

**Fig. 10:**
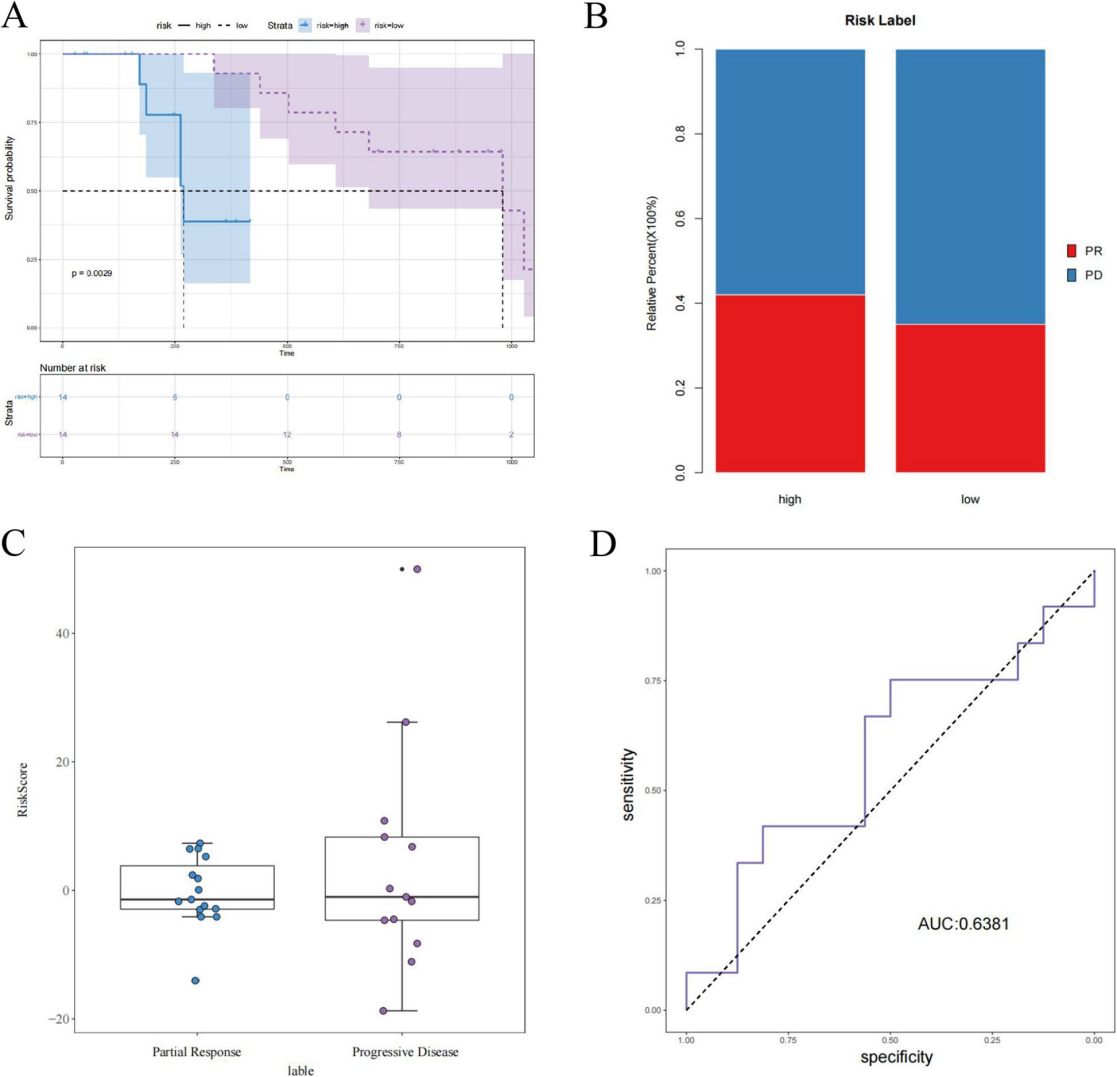
Evaluation of immunotherapy predictors for the prognostic risk signature. **A** Survival analysis. **B** Different proportions of PR and PD in high- and low-risk groups confirm the validity of the classification. **C** Risks core of PR and PD. **D** AUC curve of prognosis signature; the horizontal axis shows specificity and the longitudinal axis shows sensitivity

The prognostic risk score signature was applied to the GSE35640 (melanoma) dataset to assess whether it could be used as a predictor of immunotherapy response. As shown in Fig. 10, the PR and PD rates differed between the high- and low-risk groups, and the AUC value was greater than 0.6, indicating high efficacy (PR refers to immunotherapy predicting partial response, or effective; PD refers to immunotherapy predicting progressive disease, or non-effective).

### Sankey diagram analysis for clinical characteristics

**Fig. 11:**
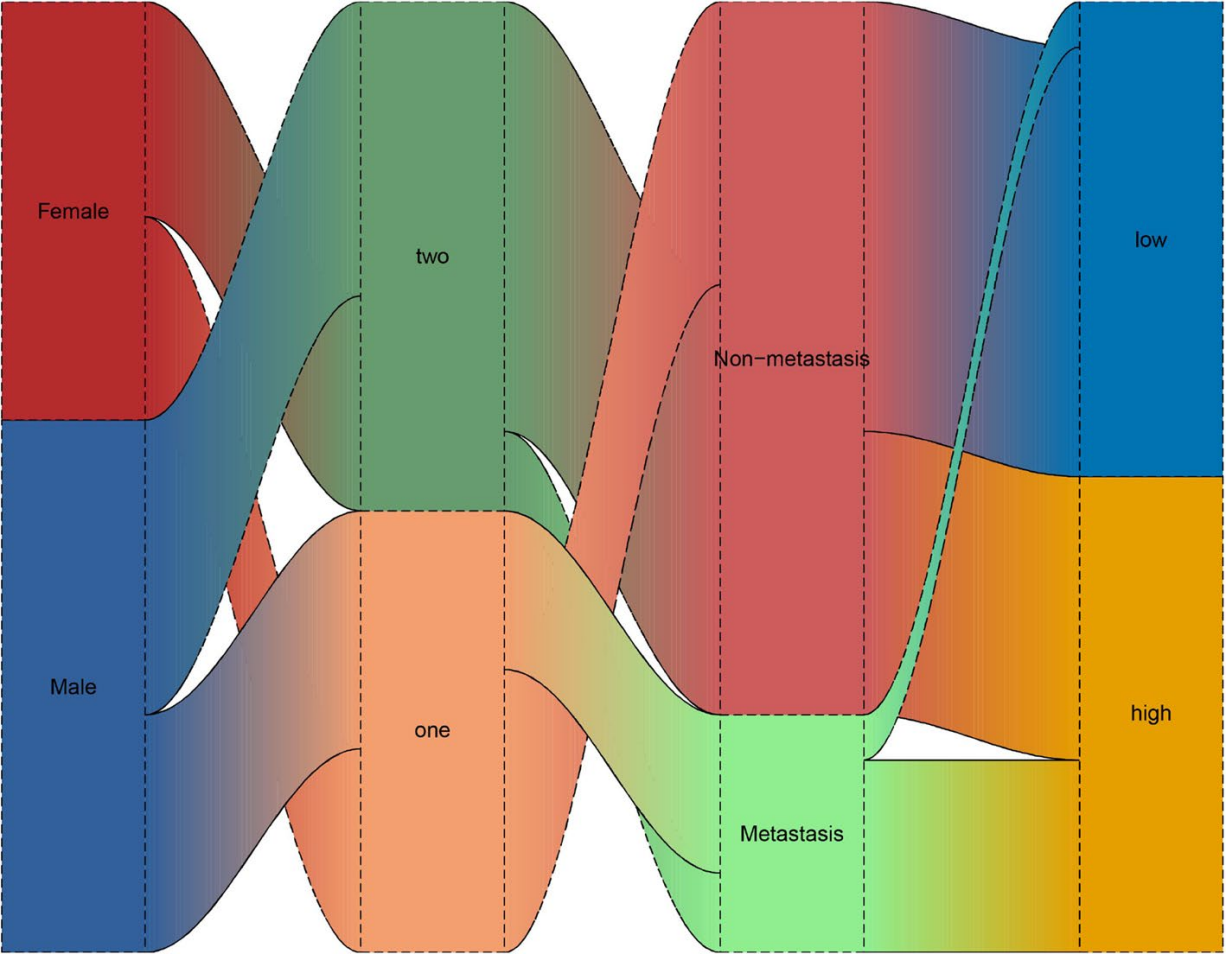
Construction of the Sankey diagram combined with clinical features

We combined the prognostic high- and low-risk groups, clustering type, and clinical characteristics to construct a Sankey diagram (Fig. 11). The Sankey diagram shows significantly differences in the proportions of patients with and without metastases in the high- and low-risk groups. Most of the high-risk patients had metastases, and only a small proportion of patients with metastases were in the low-risk group.

### Biological experimental validation on quantification of key genes in signature and malignant biological behavior of tumor cells based on these genes

**Fig. 12:**
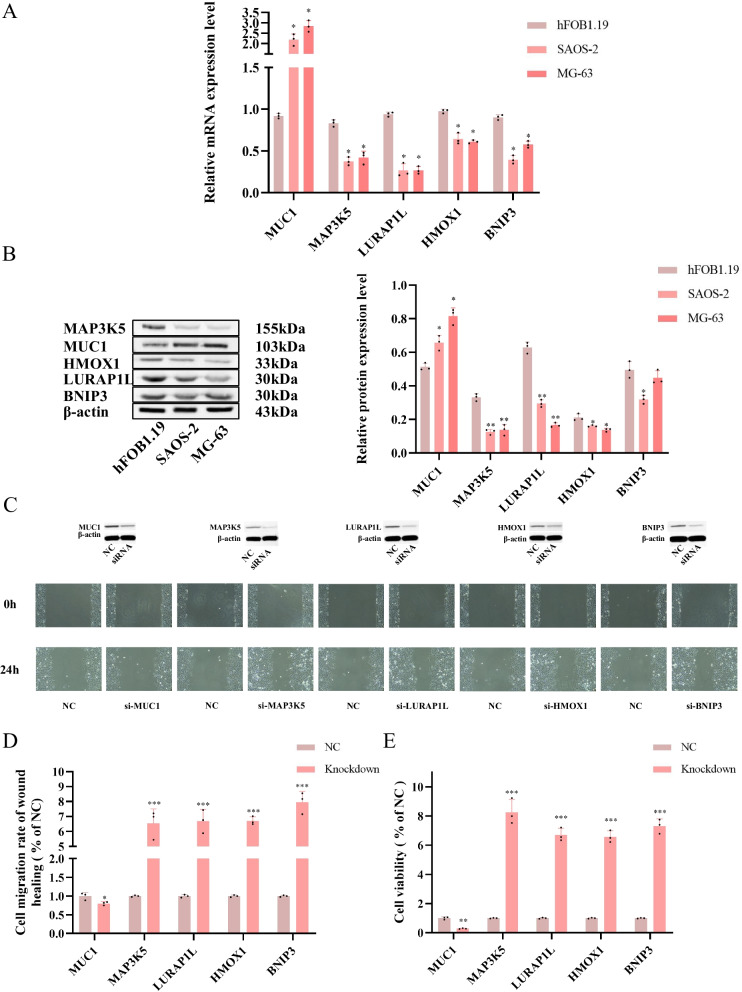
**A** The relative mRNA expression levels were measured using the fold-change in each protein relative to GAPDH. The data are expressed as the mean ± standard deviation. **B** Expression of ferroptosis-related protein was measured by western blot, The relative protein expression levels were quantified and measured using the fold-change in each protein relative to β-actin, *P < 0.05 and **P < 0.01 vs. hFOB1.19. **C** The knockdown efficiency of siRNA on five key genes was measured by westernblot, and wound healing assay results based on siRNA shows the migration ability differences. **D** Quantification of wound healing assay. **E** Cell viability assay by CCK-8. *P < 0.05, **P < 0.01, and ***P < 0.001 vs. si-NC

As Fig. 12A illustrated, the mRNA expression level of MAP3K5, LURAP1L, HMOX1 and BNIP3 decreased significantly in MG-63 and SAOS-2 cells compared with hFOB1.19 cells. MUC1 was upregulated in MG-63 and SAOS-2 cells (*P < 0.05, **P < 0.01). To further investigate the five genes in bone-derived cells, western blot (Fig. 12B) was performed to examine five ferroptosis-associated genes (MUC1, MAP3K5, LURAP1L, HMOX1, and BNIP3). MAP3K5, LURAP1L, HMOX1 and BNIP3 expression levels were markedly decreased in the osteosarcoma cells compared with the normal human osteoblast hFOB1.19 cells, they are negatively related with the riskscore. These findings revealed that each gene might characterize protective feature. However, MUC1 gene showed an obviously opposite trend and might characterize tumorigenic feature (*P < 0.05, **P < 0.01). Figure 12C showed that knockdown of gene expression by siRNA significantly inhibited the expression of five target genes. Wound healing test in SAOS-2 cell lines illustrated that knockdown of MUC1 impaired the ability of migration. On the contrary, knockdown of MAP3K5, LURAP1L, BNIP3 and HMOX1 enhanced cancer cell migration. The above results are shown quantitatively in Fig. 12D. Moreover, cell viability assays were also performed, presenting consistent tendency with the migration ability (Fig. 12E) (*P < 0.05, **P < 0.01, ***P < 0.001).

## Discussion

Current treatments for osteosarcoma include surgery and chemotherapy. Although some chemotherapeutic drugs show efficacy for the treatment of osteosarcoma, the disease control rate and survival time remain unsatisfactory. Patients with osteosarcoma have a poor prognosis, underscoring the need to explore new safe and effective treatment options in the clinic [10]. The role of ferroptosis in tumors was recently revealed and it is expected to be a new therapeutic target to guide clinical practice. Ferroptosis is a novel form of cell death caused by the massive accumulation of intracellular reactive oxygen species (ROS) and oxygen radicals. Tumor cells normally have persistently high ROS levels and are more vulnerable to ROS because of excessive oxidative stress [20]. Therefore, due to the crucial role of ROS in the initiation and progression of tumors, inducing abnormal ROS generation and accumulation might be a useful antitumor strategy [21]. Lv et al. demonstrated that PEITC induces ferroptosis, autophagy, and apoptosis in K7M2 osteosarcoma cells by activating the ROS-related MAPK signaling pathway. PEITC has promising anti-osteosarcoma activity [22, 23]. Ferroptosis is involved tumor drug resistance by impairing STAT3/Nrf2/GPX4 signaling, and it increases the sensitivity of osteosarcoma cells to cisplatin [11]. Certain pharmaceutical ingredients such as EF24, Artemisia Annua L. and Pure Artemisinin might serve as potential agents for the treatment of osteosarcoma [13, 24]. Based on theories above, ferroptosis-related factor classification and risk score signature construction is worthy of investigating.

This study analyzed downloadable osteosarcoma transcriptome data from the public database TARGET as well as 268 published ferroptosis-related genes. The samples were divided into two clusters based on the set of ferroptosis-related genes, and significant differences in survival status were detected. Functional annotation demonstrated that the differential genes were associated with inflammatory signaling pathways such as HIF-1, T cells, and IL-17. Based on inter-cluster differences in ferroptosis-related genes, prognosis-related factors were identified using univariate Cox regression analysis, and redundant factors were removed by applying LASSO regression to reduce dimensionality. Finally, five factors were selected to construct a prognostic risk score signature. The efficacy of the prognostic risk score signature was evaluated, and it was found to have better predictive efficacy than clinical characteristics such as age and gender.

In this study, five key factors in the prognostic risk-score signature were correlated with osteosarcoma. BNIP3 is a unique pro-apoptotic protein with ACAA2 as a functional binding partner in human osteosarcoma U-2OS cells [25]. Inhibition of BNIP3 expression suppresses cell apoptosis. Ye et al. showed evidence of baicalein’s anti-osteosarcoma mechanism links ROS-induced BNIP3 expression in MG-63 cells [26]. Herbert et al. showed that peptides present in secreted MUC1 may have immunoenhancing properties for osteosarcoma. As a survival-related upregulated gene in osteosarcoma, MUC1 was selected as a potential independent prognostic candidate gene, and has been associated with metastatic progression both in vivo and in vitro in several cancer types [27]. High expression of MUC1 is correlated with low survival of osteosarcoma patients [28], which is consistent with our results that MUC1 acts as a risk factor in our signature. Heme oxygenase-1 (HO-1) is a major antioxidant enzyme that plays a central role in the removal of intracellular ROS [29, 30]. Induced expression of HO-1 is responsible for the resistance of human osteosarcoma MG63 cells to the chemotherapeutic agent arsenic trioxide [31]. A study that identified four genes predicting the survival of osteosarcoma patients showed that MAP3K5 is negatively correlated with survival risk and risk score, which is consistent with our results [32]. MAP3K5 is a macrophage-associated gene signature member to predict the prognosis of osteosarcoma and might direct immunotherapy [33]. LURAP1L was identified in our risk score signature as a negative risk factor, although its role in cancer has not been reported to date. Our results indicated that knockdown of LURAP1L was in line with the increase of SAOS-2 proliferation and migration ability.

Risk score signature with five key factors illustrated in Result 5 showed that the classification efficacy and predictive efficacy of the prognostic risk score signature were better than some typical clinical characteristics, such as age, gender, survival status, occurrence of metastasis, as well as category labeling. Furthermore, we validated the prognostic risk score signature with external data, and found that it had better classification efficacy than conventional clinical features. The results of previous enrichment analysis led us to analyze differences in immune cell infiltration between high-and low- prognostic risk clusters, which showed significant infiltration differences of B cells and T cells in these subtypes. Immunotherapy has revolutionized cancer treatment and the clinical applications of immunotherapy have been adapted to range from the management of many tumors. Melanoma is characterized by rapidly spreading and life-threatening progression, which has currently the most available and comprehensive data in the public database. Therefore, melanoma datasets are used for effective verification to illustrate the advantage of the signature. Due to lack of immunotherapy dataset for osteosarcoma, we applied the prognostic risk score signature to the GSE35640 (melanoma) dataset [34] to assess whether it could be used as a predictor of immunotherapy and found that it had high efficacy as well. In future studies, this prognostic risk score signature might be the breakthrough in immunotherapy based on immunology features for cancer.

In addition, we designed in *vitro* experiments to validate the five key genes in ferroptosis-related prognosis signature. Human normal osteoblasts cell line hFOB1.19, and two kinds of human osteosarcoma cell line SAOS-2 and MG-63 are enrolled in our validation process. hFOB1.19 is the normal human osteoblast and regarded as negative control, while MG-63 and SAOS-2 are human osteosarcoma cell lines for dual validation experiments. By verifying the changes in gene expression in the three cell lines and the way in which they altered malignant biological behavior, the significance of their impact on OSA prognosis can be clarified to some extent. Based on the theory above, we examined the influence of the five key gene expression on malignant biological behavior of osteosarcoma cells, such as proliferation and migration. We used real-time PCR to initially validate the expression of five genes in three cell lines. Then it was confirmed by Western blot that the mRNA expression level of MAP3K5, LURAP1L, HMOX1 and BNIP3 decreased significantly in MG-63 and SAOS-2 cells compared with hFOB1.19 cells. MUC1 was upregulated in MG-63 and SAOS-2 cells. MAP3K5, LURAP1L, HMOX1 might serve as negative risk factor for prognosis of osteosarcoma or melanoma. Different from MUC1, knockdown of these genes could promote the proliferation and migration ability of tumor cells. While, MUC1 participated as a risk factor which is detrimental to prognosis. Aside from the four genes above, BNIP3 expression was not in concert with the role as a positive risk factor in signature. However, such results do not affect the overall guidance value of the risk score in ferroptosis-related prognosis signature. Five factors constitute the signature structure to assist in risk scoring process. However, the present study had some limitations. Additional prospective studies involving a large-cohort clinical studies are needed to confirm the role of the five ferroptosis-related signature in osteosarcoma prognosis. And further immunological features are supposed to be evaluated in pathological tissue samples in the future.

## Conclusion

Five ferroptosis-associated genes (MUC1, MAP3K5, LURAP1L, HMOX1, and BNIP3) correlated with the prognosis of osteosarcoma were screened for the construction of the risk score model. A novel ferroptosis-associated risk score signature to differentiate low- and high-risk groups of osteosarcoma was constructed based on multiple bioinformatics analyses. The signature was validated using an external independent dataset. It showed good classification efficacy for immunotherapeutic prognostic indicators for osteosarcoma and melanoma as well.

## Supplementary Information

**Additional file 1.** Supplementary materials.

**Additional file 2.** Supplementary table 1.

**Additional file 3.** Supplementary table 2.

**Additional file 4.** Supplementary table 3.

**Additional file 5.** Supplementary table 4.

## Data Availability

Publicly available datasets were analyzed in this study. This data can be found: https://ocg.cancer.gov/.

## Abbreviations

NACT: Neoadjuvant chemotherapy
TARGET: Therapeutically Applicable Research to Generate Effective Treatments
NMF: Non-negative matrix factorization
LASSO: Least absolute shrinkage and selection operator
Ferr-DEGs: Ferroptosis-related differentiated expression genes
