# Supplementary material for: Clinical significance and immune landscape of a novel ferroptosis-related prognosis signature in osteosarcoma
Source: BMC Cancer. 2023 Mar 10;23:229. doi: 10.1186/s12885-023-10688-7 (PMC10007778; doi:10.1186/s12885-023-10688-7)
Supplement: Supplementary file 1 — Additional file 1. Supplementary materials. [file 12885_2023_10688_MOESM1_ESM.pdf]

Supplementary Material

Raw blot images of each western blot data mention in figure 12B. Blots were cut prior to hybridisation with antibodies and all replicates are performed, and the raw data of the electronic version of the cut-out figure element are retained.

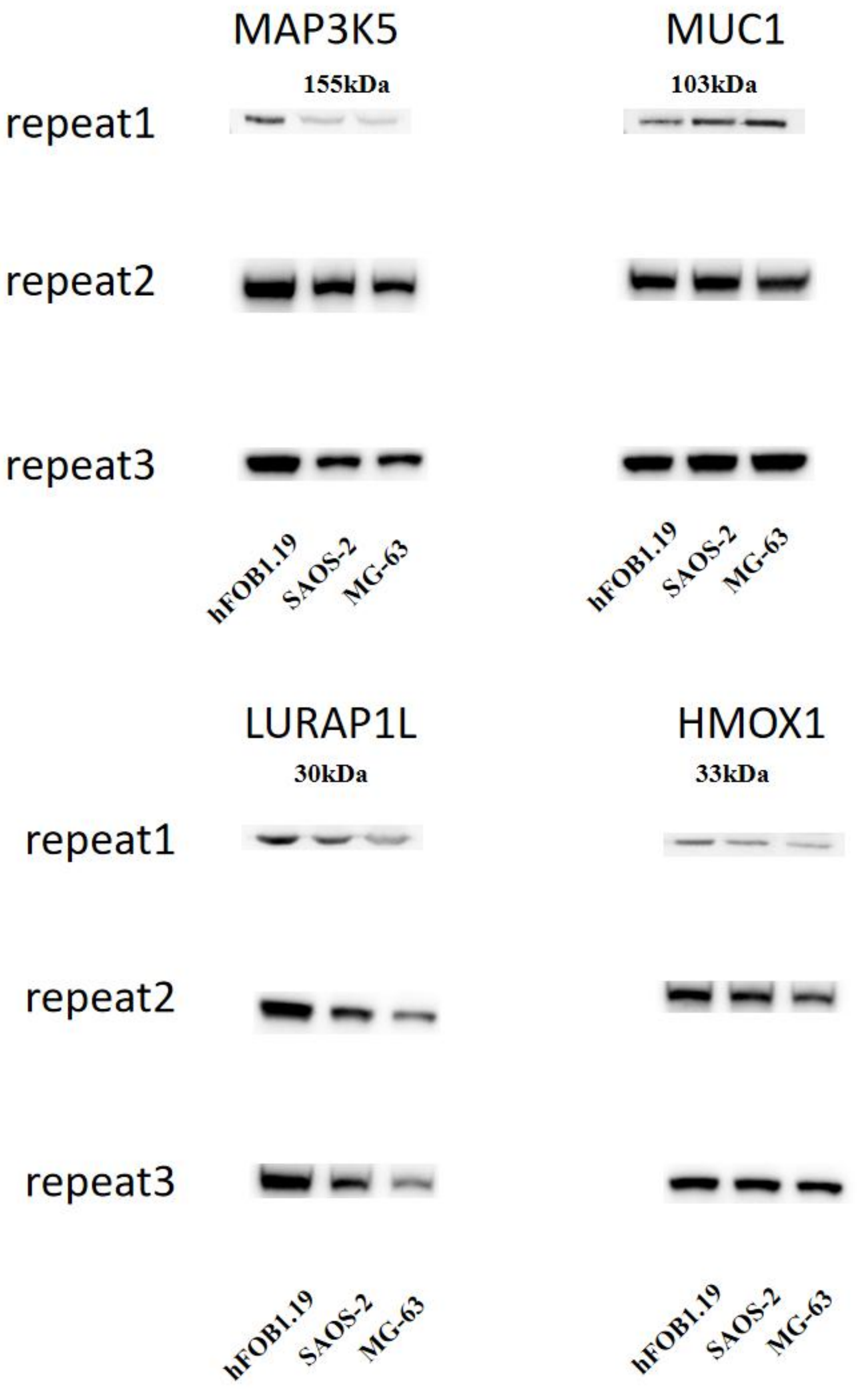

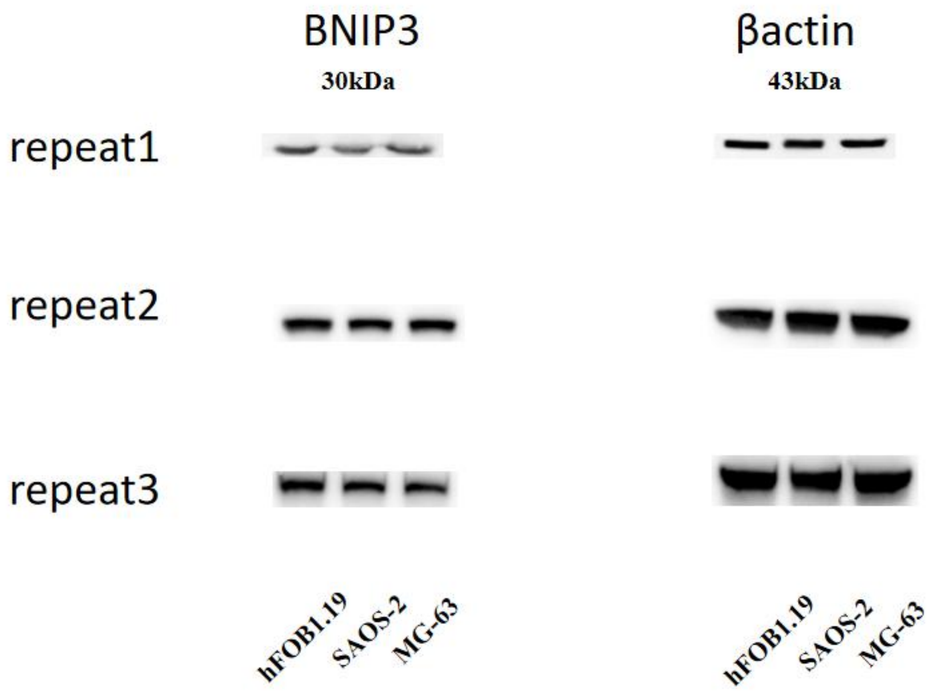

Figure 12C

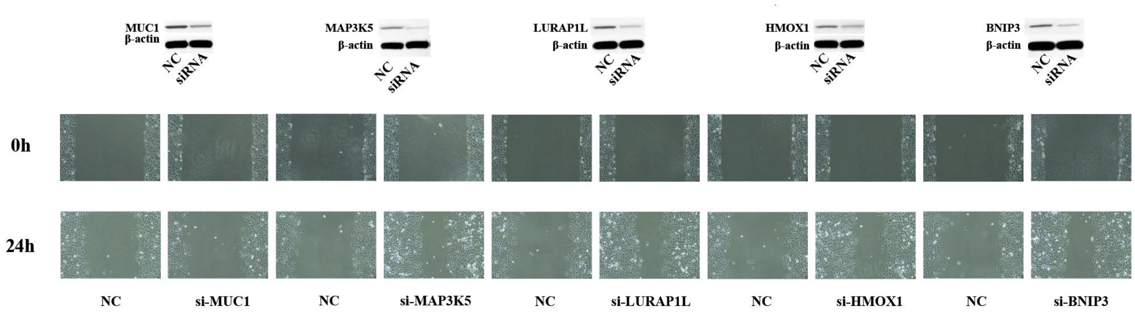

Figure 12D

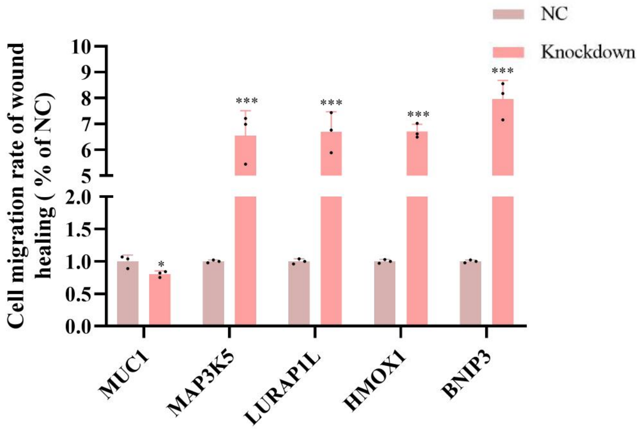
NC
Knockdown
Cell migration rate of wound healing (% of NC)
10
9
8
7
6
5
2.0
1.5
1.0
0.5
0.0
*
***
***
***
***
MUC1
MAP3K5
LURAP1L
HMOX1
BNIP3

Figure 12E

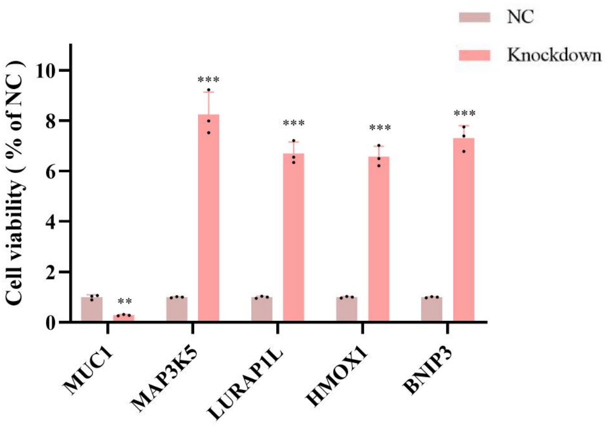
NC
Knockdown
Cell viability (% of NC)
10
8
6
4
2
0
**
***
***
***
***
MUC1
MAP3K5
LURAP1L
HMOX1
BNIP3
